# Supplementary material for: Methicillin resistance Staphylococcus aureus nasal carriage and its associated factors among HIV patients attending art clinic at Dessie comprehensive specialized hospital, Dessie, North East Ethiopia
Source: PLOS Glob Public Health. 2022 Sep 12;2(9):e0000838. doi: 10.1371/journal.pgph.0000838 (PMC10021588; doi:10.1371/journal.pgph.0000838)
Supplement: Attachment — Submitted filename: response to reviwers.docx [file pgph.0000838.s001.docx]

PGPH-D-22-00354
Methicillin Resistance Staphylococcus aureus Nasal Carriage and Its Associated Factors Among HIV Patients Attending ART Clinic at Dessie Comprehensive Specialized Hospital, Dessie, North East Ethiopia
PLOS Global Public Health

Thank you for submitting your manuscript to PLOS Global Public Health. After careful consideration, we feel that it has merit but does not fully meet PLOS Global Public Health’s publication criteria as it currently stands. Therefore, we invite you to submit a revised version of the manuscript that addresses the points raised during the review process.

Please submit your revised manuscript by . If you will need more time than this to complete your revisions, please reply to this message or contact the journal office at [globalpubhealth@plos.org](mailto:globalpubhealth@plos.org). When you're ready to submit your revision, log on to <https://www.editorialmanager.com/pgph/> and select the 'Submissions Needing Revision' folder to locate your manuscript file.

Please include the following items when submitting your revised manuscript:

- A rebuttal letter that responds to each point raised by the editor and reviewer(s). You should upload this letter as a separate file labeled 'Response to Reviewers'.
- A marked-up copy of your manuscript that highlights changes made to the original version. You should upload this as a separate file labeled 'Revised Manuscript with Track Changes'.
- An unmarked version of your revised paper without tracked changes. You should upload this as a separate file labeled 'Manuscript'.

Journal Requirements:

1. Your co-authors, Hussein Muhaba ([labhussein@yahoo.com](mailto:labhussein@yahoo.com)) and Genet Molla Fenta ([mgenetaskalu@gmail.com](mailto:mgenetaskalu@gmail.com)), have not confirmed authorship of the manuscript. We have resent them the authorship confirmation email; however please check that the above email address for them is correct and follow up personally to ensure they confirm. Please note that we cannot pass your manuscript to Production until we have received confirmations from all co-authors.

Just in case your co-authors are having difficulty confirming their authorship, you may advise them to send us an email at [globalpubhealth@plos.org](mailto:globalpubhealth@plos.org) and we will confirm their authorship on the authors' behalf.

**Author response**: I have informed them and they will provide authorship confirmation very soon.

2. Please provide a detailed Financial Disclosure statement. This is published with the article, therefore should be completed in full sentences and contain the exact wording you wish to be published.

i) Please include all sources of funding (financial or material support) for your study. List the grants (with grant number) or organizations (with url) that supported your study, including funding received from your institution. 
ii). State the initials, alongside each funding source, of each author to receive each grant.
iii). State what role the funders took in the study. If the funders had no role in your study, please state: “The funders had no role in study design, data collection and analysis, decision to publish, or preparation of the manuscript.”
iv). If any authors received a salary from any of your funders, please state which authors and which funders.

If you did not receive any funding for this study, please simply state: “The authors received no specific funding for this work.”

**Author response**: Since we did not received fund for this study we have stated “The authors received no specific funding for this work” in the manuscript.

3. Please update your Competing Interests statement. If you have no competing interests to declare, please state: “The authors have declared that no competing interests exist.”

**Author response**: We have made the necessary correction

4. In the online submission form, you indicated that “All Data are available up on proper request”. All PLOS journals now require all data underlying the findings described in their manuscript to be freely available to other researchers, either 1. In a public repository, 2. Within the manuscript itself, or 3. Uploaded as supplementary information.
This policy applies to all data except where public deposition would breach compliance with the protocol approved by your research ethics board. If your data cannot be made publicly available for ethical or legal reasons (e.g., public availability would compromise patient privacy), please explain your reasons by return email and your exemption request will be escalated to the editor for approval. Your exemption request will be handled independently and will not hold up the peer review process, but will need to be resolved should your manuscript be accepted for publication. One of the Editorial team will then be in touch if there are any issues.

**Author response**: All data are available in the manuscript and I can provide if you want to see the questionnaire or any other data like the SPSS raw data and so on. Let me know what kind of data you want to see.

5. We do not publish any copyright or trademark symbols that usually accompany proprietary names, eg (R), (C), or TM (e.g. next to drug or reagent names). Therefore please remove all instances of trademark/copyright symbols throughout the text, including Male®, 60®, Urban®, Single®, No®, 500®, First®, College and above®, No contact®, Undetected®, and ®; reference category.

**Author response**: We have removed all of these unnecessary symbols

6. Please review your reference list to ensure that it is complete and correct. If you have cited papers that have been retracted, please include the rationale for doing so in the manuscript text, or remove these references and replace them with relevant current references. Any changes to the reference list should be mentioned in the rebuttal letter that accompanies your revised manuscript. If you need to cite a retracted article, indicate the article’s retracted status in the References list and also include a citation and full reference for the retraction notice.

**Author response**: The reference list is complete and correct. We have not used the retracted article

Additional Editor Comments (if provided):

Minor Correction is needed. Please do the needful correction.


[Note: HTML markup is below. Please do not edit.]

Reviewers' comments:

Reviewer's Responses to Questions

**Comments to the Author**

1. Does this manuscript meet PLOS Global Public Health’s [publication criteria](https://journals.plos.org/globalpublichealth/s/criteria-for-publication)? Is the manuscript technically sound, and do the data support the conclusions? The manuscript must describe methodologically and ethically rigorous research with conclusions that are appropriately drawn based on the data presented.

Reviewer #1: Yes

Reviewer #2: Yes

2. Has the statistical analysis been performed appropriately and rigorously?

Reviewer #1: Yes

Reviewer #2: Yes

3. Have the authors made all data underlying the findings in their manuscript fully available (please refer to the Data Availability Statement at the start of the manuscript PDF file)?

The [PLOS Data policy](https://journals.plos.org/globalpublichealth/s/data-availability) requires authors to make all data underlying the findings described in their manuscript fully available without restriction, with rare exception. The data should be provided as part of the manuscript or its supporting information, or deposited to a public repository. For example, in addition to summary statistics, the data points behind means, medians and variance measures should be available. If there are restrictions on publicly sharing data—e.g. participant privacy or use of data from a third party—those must be specified.

Reviewer #1: Yes

Reviewer #2: Yes

4. Is the manuscript presented in an intelligible fashion and written in standard English?

PLOS Global Public Health does not copyedit accepted manuscripts, so the language in submitted articles must be clear, correct, and unambiguous. Any typographical or grammatical errors should be corrected at revision, so please note any specific errors here.

Reviewer #1: Yes

Reviewer #2: Yes

5. Review Comments to the Author

Please use the space provided to explain your answers to the questions above. You may also include additional comments for the author, including concerns about dual publication, research ethics, or publication ethics. (Please upload your review as an attachment if it exceeds 20,000 characters)

Reviewer #1:

Abstract: Factors related to the association of MRSA nasal carriage among HIV patients should be mentioned in the result section of the abstract.

**Authors Response**: Three factors (Having job close contact with human, picking the nose and ART failure) were already mentioned in the original manuscript but we did not put the statistical value. So, we made the necessarily modification in this section.

Introduction: This section is nicely written. The association factor that has been written in the title was not described in the introduction. However, rationally should be written more precious and elaborately.

**Author response**: Information about the associated factors was mentioned in the original manuscript in line 74-76. But to make it more elaborative we added some important information according to your comment.

Methods: Sample Size determination(Line 101): please write sample size calculation in text form instead of formula.

**Author response:** Thank you for the comment and we made the required adjustment.

Sample collection(Line 135): has been properly performed by the Triple package system (Line 139).

**Author response:** Thanks again for your constructive comment and suggestion. We made appropriate correction.

Culture (Line 156): Why Mac Conkey's media is used?

**Author Response**: As you can see from Table 3 we have also tried to detect the nasal colonization of gram negative rods. So, in order to do so we have to use Mac Conkey media.

Ethics statement(Line 192): from where ethical clearance was taken? please mention it with the number.

**Author response**: Thank you for the comments. We have merged the information in Ethical statement and Ethics approval section in the original manuscript and we called it now Ethical consideration. So, the reader can get all the necessary information about ethical clearance providing agent, Ethical approval number and so on.

Ethics approval (Line 375): This section is nicely written with mentions proper information.

**Author Response**: Thank you

Conclusion: Avoid Line 346 &347. It is repetition. The conclusion should be written in the present tense.

**Author Response**: Thanks for the comment and based on your suggestion we made the necessary modification.

Table: Avoid the symbol of percentage % in the heading of the table.

**Author response**: Thanks for the comment and we have taken the appropriate correction as per your suggestion

The author has said that in the result section Table 2 about CD4 count is baseline & recent. But this has not been mentioned in the methodology section. The procedure of measuring CD4 count is missing.

**Author response**: You are right, we have mentioned about the baseline and recent CD4 count in the result section but we do not mentioned in the methodology section. This is because we did not perform the test rather we have taken the information from the patients hospital record.

Table4: Does it duplicate with Table1?

**Author response**: No, Table 1 provide information about the socio-demographic data whereas, Table 4 indicates the distribution of nasal colonization of *S. aureus* and MRSA in line with the socio-demographic data.

Table5: Why age>60 years is considered as a reference?

**Author response**: Different articles mentioned study subjects whose age greater than 60 and less than 30 years are at risk of acquiring the MRSA colonization. We choose age greater than 60 years as a reference, this is because of difference in the number of participants in each of the above age category 20 (< 30 years) and 14 (>60 years).

Table5 & Table6:is it the repetition?

**Author response**: No, Table 5 represents the bivariable and multivariable analysis of *S. aureus* colonization whereas; Table 6 demonstrated the bivariable and multivariable analysis of MRSA colonization. Look at the associated factors, there are differences between S. aureus and MRSA colonization

Table7: Mention either sensitive or resistant pattern. please avoid both.

# Author response: Thanks for the comment. We want to describe about the antibiotic susceptibility (it can be sensitive or resistance) pattern of the isolate. So it is appropriate to use both sensitive as well as resistance rate. We have tried to see various published articles in this area and most of them used both sensitive and resistance rate.

# Look at the following articles:

# Gebremedhn et al., 2016 Prevalence and risk factors of methicillin-resistant *Staphylococcus aureus* colonization among HIV patients in Mekelle, Northern Ethiopia

- Kalash Neupane et al., 2018. Comparison of Nasal Colonization of Methicillin-Resistant *Staphylococcus aureus* in HIV-Infected and Non-HIV Patients Attending the National Public Health Laboratory of Central Nepal. [Canadian Journal of Infectious Diseases and Medical Microbiology](https://www.hindawi.com/journals/cjidmm/)

Reviewer #2: The rate of S. aureus and MRSA nasal colonization was high and it has associated with different factors. Understanding and managing MRSA among HIV patients is mandatory and stakeholders should find out the way how to decolonize the bacteria from nasal area, SO it was a nice paper.

**Author response**: Thanks for your opinion regarding the current manuscript and for the topic at all. You are right it needs a collaborative effort to reduce the rate of colonization. Unless and otherwise the colonization among HIV subjects might result in life threatening effect.

6. PLOS authors have the option to publish the peer review history of their article ([what does this mean?](https://journals.plos.org/globalpublichealth/s/editorial-and-peer-review-process#loc-peer-review-history)). If published, this will include your full peer review and any attached files.

**Do you want your identity to be public for this peer review?** If you choose “no”, your identity will remain anonymous but your review may still be made public.


For information about this choice, including consent withdrawal, please see our [Privacy Policy](https://www.plos.org/privacy-policy).

Reviewer #1: No

Reviewer #2: **Yes:** Arifa Akram
