# Supplementary material for: Methicillin resistance Staphylococcus aureus nasal carriage and its associated factors among HIV patients attending art clinic at Dessie comprehensive specialized hospital, Dessie, North East Ethiopia
Source: PLOS Glob Public Health. 2022 Sep 12;2(9):e0000838. doi: 10.1371/journal.pgph.0000838 (PMC10021588; doi:10.1371/journal.pgph.0000838)
Supplement: Attachment — Submitted filename: Response to Reviewers.docx [file pgph.0000838.s002.docx]

**REBUTTAL LETTER**

We would like to thank the editor and the reviewer (s) for their comments on our manuscript title “Methicillin Resistance *Staphylococcus aureus* Nasal Carriage and Its Associated Factors among HIV Patients Attending ART Clinic at Dessie Comprehensive Specialized Hospital, Dessie, North East Ethiopia.” Below is our response to point raised by the academic editor and reviewer (s). We hope that we satisfyingly addressed them and that the manuscript will be now suited for publication.

Sincerely,

On behalf of all authors,

Daniel Gebretsadik Weldehanna

1. Please review your reference list to ensure that it is complete and correct. If you have cited papers that have been retracted, please include the rationale for doing so in the manuscript text, or remove these references and replace them with relevant current references. Any changes to the reference list should be mentioned in the rebuttal letter that accompanies your revised manuscript. If you need to cite a retracted article, indicate the article’s retracted status in the References list and also include a citation and full reference for the retraction notice.

**Author response**: We have checked the reference list in this manuscript carefully and we obtained one reference (reference 40) that has **thesis reference type**. We have changed this reference with other similar journal article. We have checked all the 42 references that are found in the reference list but we did not find any retracted reference list in this particular manuscript.
