# Supplementary material for: Methicillin resistance Staphylococcus aureus nasal carriage and its associated factors among HIV patients attending art clinic at Dessie comprehensive specialized hospital, Dessie, North East Ethiopia
Source: PLOS Glob Public Health. 2022 Sep 12;2(9):e0000838. doi: 10.1371/journal.pgph.0000838 (PMC10021588; doi:10.1371/journal.pgph.0000838)
Supplement: Attachment — Submitted filename: Second response to editorial team.docx [file pgph.0000838.s003.docx]

**REBUTTAL LETTER**

We would like to thank the editor and the reviewer (s) for their comments on our manuscript title “Methicillin Resistance *Staphylococcus aureus* Nasal Carriage and Its Associated Factors among HIV Patients Attending ART Clinic at Dessie Comprehensive Specialized Hospital, Dessie, North East Ethiopia.” Below is our response to point raised by the academic editor and reviewer (s). We hope that we satisfyingly addressed them and that the manuscript will be now suited for publication.

Sincerely,

On behalf of all authors,

Daniel Gebretsadik Weldehanna

- Please explain what MDR is at first it is written.

**Author response**: Thanks for the comment, at this time we have taken the necessarily correction.

- "..Ethiopia reported a lower prevalence (6)" what is their findings?

**Author response**: Thanks for your query of further clarification. According to their report the *S. aureus* colonization rate was 39.7%, the prevalence of MRSA among *S. aureus*-positive HIV patients was 52.45% (64/122) and the overall prevalence of MRSA among HIV patients was 20.8% (64/307)

- For "Screening of Methicillin-Resistant S. aureus", did authors used positive and negative control strains. ?

**Author response**: Thanks for the question. We have used both Methicillin-Resistant and Methicillin-Susceptible *S. aureus* strains as a control in the screening of MRSA.

- Why authors did not use any molecular analysis to identify the strains as *S. aureus*.

**Author response**: Thanks for the question. During the study period there was no facility for molecular techniques (analysis) in the study area and we could not get the access in other places, although we have tried.

- Any control strains used for antimicrobial study?

**Author response**: Thanks for the question. We have used the control strain for the testing of antimicrobial and we have described in the methodology section of the manuscript.
