# Supplementary material for: Methicillin resistance Staphylococcus aureus nasal carriage and its associated factors among HIV patients attending art clinic at Dessie comprehensive specialized hospital, Dessie, North East Ethiopia
Source: PLOS Glob Public Health. 2022 Sep 12;2(9):e0000838. doi: 10.1371/journal.pgph.0000838 (PMC10021588; doi:10.1371/journal.pgph.0000838)
Supplement: Attachment — Submitted filename: Third Response.docx [file pgph.0000838.s004.docx]

**REBUTTAL LETTER**

We would like to thank the editor of the Journal for all helpful comments on our manuscript title “Methicillin Resistance *Staphylococcus aureus* Nasal Carriage and Its Associated Factors among HIV Patients Attending ART Clinic at Dessie Comprehensive Specialized Hospital, Dessie, North East Ethiopia.” Below is our response to point raised by the academic editor. We hope that we satisfyingly addressed them and that the manuscript will be now suited for publication.

Sincerely,

On behalf of all authors,

Daniel Gebretsadik Weldehanna

Journal Requirements:
1. Please review your reference list to ensure that it is complete and correct. If you have cited papers that have been retracted, please include the rationale for doing so in the manuscript text, or remove these references and replace them with relevant current references. Any changes to the reference list should be mentioned in the rebuttal letter that accompanies your revised manuscript. If you need to cite a retracted article, indicate the article’s retracted status in the References list and also include a citation and full reference for the retraction notice.

**Author’s response**: Thanks for your positive comment. We have made few changes in the reference list. Reference 25 in the previous document was the 2011 CLSI guideline (which was editing error) but we have corrected in the current manuscript (we have mentioned CLSI 2019 guideline).

Additional Editor Comments (if provided):

Dear Author, thank you very much for your revision. I just would like to ask some further revisions, i) in material and method section, there is no need to write a quality control paragraph, so that just put control strain into necessary sections, and also add the ATCC number for strains (both resistant and sensitive).

# Author’s response: Thank you for the comment and suggestion. The positive control strain for *S. aureus* is ATCC–25923 in all microbiological laboratory analysis: isolation, identification and drug susceptibility. The screening for MRSA and MSSA identification was done by using cefoxitin (30 *µ*g) antibiotic disc following modified Kirby-Bauer disc diffusion technique. The *S. aureus* zone size of ≥ 22 mm around cefoxitin disk was confirmed as MSSA strain and ≤21 mm around cefoxitin disk was confirmed as MRSA strain as per the CLSI 2019 guidelines. Different articles also used the same strain for MRSA and MSSA isolation, the criteria to distinguish MRSA strains from MSSA strains is the zone of inhibition diameter against cefoxitin disk. In addition, *Escherichia coli* (ATCC 25922) strain was also used as a negative control.
